# Recombination and selection in the major histocompatibility complex of the endangered forest musk deer (*Moschus berezovskii*)

**DOI:** 10.1038/srep17285

**Published:** 2015-11-25

**Authors:** Ruibo Cai, Aaron B.A. Shafer, Alice Laguardia, Zhenzhen Lin, Shuqiang Liu, Defu Hu

**Affiliations:** 1 College of Nature Conservation, Beijing Forestry University, China; 2 Department of Ecology and Genetics, Uppsala University, Sweden; 3 Key Laboratory of Animal Ecology and Conservation Biology, Institute of Zoology, Chinese Academy of Sciences, Beijing, China

## Abstract

The forest musk deer (*Moschus berezovskii*) is a high elevation species distributed across western China and northern Vietnam. Once abundant, habitat loss and poaching has led to a dramatic decrease in population numbers prompting the IUCN to list the species as endangered. Here, we characterized the genetic diversity of a Major Histocompatibility Complex (MHC) locus and teased apart driving factors shaping its variation. Seven *DRB* exon 2 alleles were identified among a group of randomly sampled forest musk deer from a captive population in the Sichuan province of China. Compared to other endangered or captive ungulates, forest musk deer have relatively low levels of MHC genetic diversity. Non-synonymous substitutions primarily occurred in the putative peptide-binding region (PBR), with analyses suggesting that recombination and selection has shaped the genetic diversity across the locus. Specifically, inter-allelic recombination generated novel allelic combinations, with evidence for both positive selection acting on the PBR and negative selection on the non-PBR. An improved understanding of functional genetic variability of the MHC will facilitate better design and management of captive breeding programs for this endangered species.

Characterizing patterns of diversity in functionally relevant genes can help tease apart the relative influence of genetic drift, mutation, recombination, and selection. Specifically understanding how such functional variation is generated and maintained within populations of endangered species is a central issue in conservation biology with numerous applications^1^. The importance of genetic diversity at markers of adaptive significance, such as the major histocompatibility complex (MHC) in vertebrates has long been recognized^2^,^3^. MHC variants have been shown to influence many aspects of an individual’s biology, most notably antigen recognition and susceptibility to infectious diseases, but also mating preferences and kin recognition^4^,^5^. Accordingly, MHC genes are considered a prime example of adaptive evolution and a key measure of genetic health when it comes to species conservation^6^,^7^.

The MHC is responsible for antigen-presenting proteins. Class I MHC genes primarily respond to intracellular antigens, while Class II products bind to extracellular antigens. Class III genes code for proteins participating in the complement cascade reaction^8^. The Class II genes, due to their role in fending off environmental pathogens and parasites, have received the most attention^4^. Three designated MHC class II genes have been described in humans: *DP, DQ* and *DR*. The *DR* gene is highly polymorphic and recognizes to a wide variety of antigens. In ruminant species such as cattle and sheep, only two MHC *class II* genes are observed (*DQ* and *DR*)^9^.

MHC gene diversity is generated and maintained by point mutations and balancing selection, combined with translocations and gene duplications^10^. Because of the high levels of diversity, recombination – both inter and intra-allelic – is also thought to be operating on the generation of novel MHC alleles^5^,^11^,^12^ and facilitating adaptive evolution^13^. It has been proposed that MHC genes, due to their important biological functions, should play a role in the design of programs to conserve genetic diversity in captive populations of endangered species, and generally be maintained in natural populations^14^,^15^. Thus, characterizing the levels of genetic variability and exploration on mechanisms maintaining such diversity at MHC loci are prerequisites if such information is to be included when designing captive breeding and management programs. Further, by borrowing assays developed in closely related species (i.e. genome enabled^2^), the ability to screen functional diversity in wild, often rare and endangered populations has been greatly enhanced.

One species that could benefit from such investigations is the endangered forest musk deer (*Moschus berezovskii*). China encompasses the primary range of the forest musk deer, but poaching and habitat loss have had a severe impact. The population has declined dramatically in the past five decades, prompting an endangered listing by the International Union for Conservation of Nature (IUCN^16^), Appendix I designation by the Convention on International Trade in Endangered Species of Wild Fauna and Flora (CITES 2000), and a National Class I protected species designation in China. Forest musk deer in the wild are estimated to number between 100,000–200,000^17^, with this number declining^16^. At present, approximately 7,000 forest musk deer are kept in *pseudo* captivity^18^. These programs are important for both conservation efforts and economic profit because the musk produced by males is used in Chinese and Vietnamese medicines (and is the driver behind the illegal harvest).

Previous studies have described the variation of MHC *DRB* genes of forest musk deer^19^,^20^, but these data are not in public data repositories nor have been examined in a broader context (i.e. estimate species wide functional genetic diversity). In this study, we genotyped the MHC class II locus in a captive forest musk deer population, and analyzed patterns of genetic diversity and molecular evolution of *DRB* exon 2, specifically focusing on inferring how recombination and selection shape genetic diversity. We compared our diversity data to additional species to put our data in context with other ungulate MHC diversity estimates, and discussed the conservation and evolutionary potential of forest musk deer.

## Results

Applying the criterion that an allele must be observed in at least two independent PCRs, we detected seven unique alleles in musk deer with six being present in multiple individuals (Genbank Accession nos: KP763631-KP763636, KP835297), with one haplotype (*Mobe*-DRB*04) contained deletion. We only ever observed a maximum of two alleles per individual, suggestion that only one locus was amplified.

**Figure 1:**
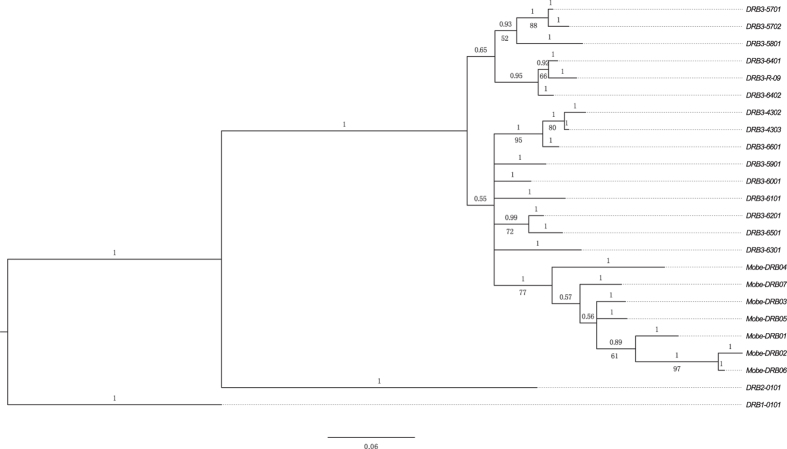
Phylogenetic tree of *DRB* exon 2 sequences (249 bp) of forest musk deer and 15 Bola *DRB3* sequences from bovine MHC *DRB3* loci (GenBank Accession nos: AY817099–AY817102, AY817104–AY817106, AY826404–AY826411). The BoLA DRB1*0101 (GenBank Accession nos: M30009) was used to root the tree. The numbers over the branch indicate Bayesian posterior probabilities while the numbers below the branch are the likelihood bootstrap values (only those >50% are shown).

The allelic sequences of 249 bp of *DRB* exon 2 were aligned and translated into the corresponding amino acid sequences. No amino acid sequence showed evidence of a reading-frame shift or stop codons. *DRB* sequences of forest musk deer along with standard *Bos taurus DRB* MHC loci (*Bola DRB*1, *DRB*2, *DRB*3) were included to examine whether all of the *Mobe DRB* sequences were derived from the same locus. The phylogenetic trees showed that all of the *Mobe DRB* alleles fell into the *DRB*3 category (Fig. 1).

### Sequence variation

**Figure 2:**
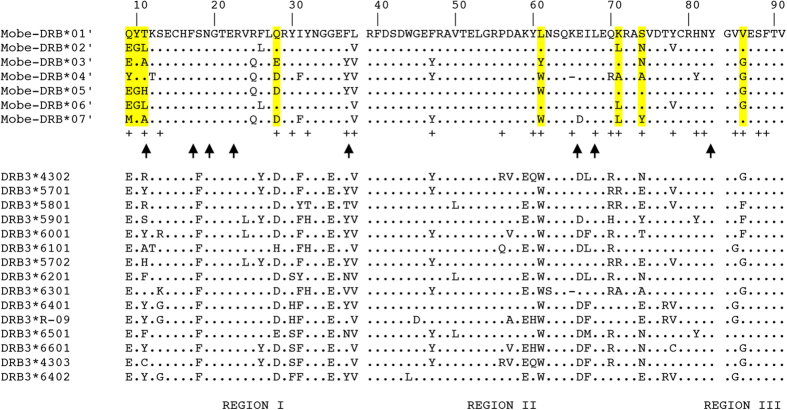
Alignment of the amino acid sequences of residues 9 to 91 of the first domain of *DRB* alleles of forest musk deer. “+” indicates residues in the peptide binding region according to human *DRB* structure^47^ and residues highlighted in yellow show positively selected sites. Arrows indicate postulated recombination breakpoints. The major configurations of the protein are shown: β-strand region (REGION I), the major part of α-helical region (REGION II), and the carboxyterminal part of the α-helical region (REGION III).

At the sequence level, 43 of the 249 nucleotides (17%) were variable; 19 amino acid residues out of 83 (23%) were polymorphic. Thirteen of the 19 variable amino acid sites (68%) were found within the putative PBR (Fig. 2). Compared to the bovine *DRB*3 loci in our analysis, there was relatively low diversity observed in the forest musk deer (Table 1).

### Recombination and selection analysis

**Figure 3:**
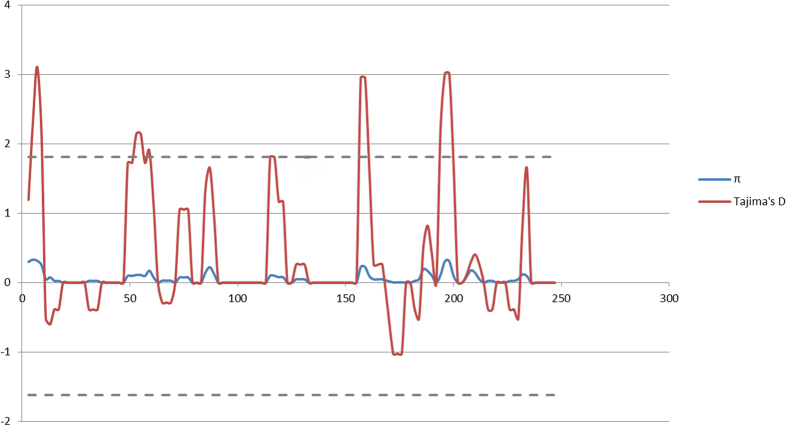
Sliding windows plots of Tajima’s D, and nucleotide diversity, *π*, are shown across the entire *DRB* exon 2. The dashed lines indicate the upper and lower limits of 95% confidence interval for Tajima’s D.

Under the assumption of orthology, we detected between 1 and 4 recombination events (Fig. 2 and Table S2). The relative rates of non-synonymous (dN) and synonymous (dS) are shown in Table 2: notably, the ratio of dN to dS was 1.88 in the PBR, and 0.31 in the non-PBR. Across the entire exon, we detected a positive Tajima’s D (2.01; Table 1). Visual characterizing via a sliding-window analysis of *π* and Tajima’s D suggested that *Mobe DRB* locus is under both positive and negative selection pressures (Fig. 3). We found 8 residues showing evidence of positive selection (Fig. 2), mostly corresponding to the location of the putative PBR (there are 24 residues within the putative PBR in humans^21^). Accounting for recombination, the selection tests produced differing results; notably, all of three methods taking into consideration the presence of recombination detected fewer residues under positive selection (Tables S4 and S5).

## Discussion

We assayed the MHC diversity in an endangered population of forest musk deer, showing that positive selection and recombination has contributed to the contemporary diversity. While the genetic signatures are consistent with selection, these could be confounded the by the demographic history (i.e. a bottleneck also produces a positive Tajima’s D value), or a combination of both. Visually assessing the *DRB* amino acid structure of Li *et al.*^19^, we found that our samples contain 5 of the 6 alleles observed in that population, suggesting we have captured a large portion of the MHC diversity seen in the species for this region. This is an important observation as the wild population numbers are declining, and captive populations are suffering outbreaks of disease^22^, and thus characterizing and understanding the driver behind MHC diversity is critical for this species.

We observed seven *Mobe DRB* exon 2 alleles in the forest musk deer (two different from what was observed in Li *et al.*^19^ that screened 65 individuals). While comparisons among species are confounded by a multitude of factors (i.e. demographic and phylogeographic histories, spatial and temporal sampling), they can be useful for identifying broader trends in MHC diversity^23^, and in the case of musk deer, assaying the relative diversity of an important functional gene where diversity is thought to harbor benefits is clearly informative. When compared with other studies on endangered or bottlenecked ungulates (see Table 3), forest musk deer were similar to Spanish ibex and chamois^24^,^25^, more diverse than Arabian oryx and mountain goat^26^,^27^, but less diverse than white-tailed deer and bighorn sheep^28^,^29^. The latter two species are common, relatively widely distributed ungulates in North America, so this relative ranking of diversity makes some biological sense. However, other immune genes have been shown to harbor high levels of diversity when the *DRB* locus appears depauperate^27^, thus it is possible that forest musk deer genetic diversity is higher among other immune-relevant genes.

Collectively, our study and that of Li *et al.*^19^ suggests the haplotype diversity we found reflect broader patterns of species diversity, at least in Sichuan province. It is reasonable to believe that the lack of extensive polymorphism at the *DRB* locus is due to the initial founding event of the captive population and/or a historical population decline (as would be supported by the positive Tajima’s D). Regardless of the driving factor, this relative low level of variation in its adaptive immune system might make this species more susceptible to disease outbreaks^20^.

Numerous mechanisms help generate diversity at the MHC loci^5^,^12^,^30^. The higher rate of non-synonymous substitutions, we suggest, are likely in response to co-evolution with exogenous antigens as the polymorphisms were predominantly located in the PBR. We also found evidence for multiple (intra-allelic) recombination events creating novel *DRB* haplotypes. While the methods varied in their conservative nature, the breakpoints at position 87 and 221 were detected by all seven approaches are close to the known connection sites (position 88 to 93, and position 223 to 228) between three domains of β-strand region, the major part of α-helical region, and the carboxyterminal part of α-helical region 9 (Fig. 2). We hypothesize that the differences in secondary structure might produce a recombination hotspot in this region of the MHC. Furthermore, our estimated population scaled recombination rate (ρ = 11.28) and mutation rate (θ = 13.88) revealed that recombination and mutation together function on the generation of *Mobe DRB* diversity (ρ/θ = 0.81), which is similar to observations made on other ungulates^31^. It is worth noting that *Mobe*-DRB*04′ contains a codon-deletion, which has also been found in both bovine and cervid *DRB* genes^32^, and the breakpoint found directly at the codon-deletion site (position 171) suggests inter-allelic recombination^32^.

Fitness-related genes, such as MHC genes, are often under a variety of selection pressures^33^. Previous studies concluded that balancing selection maintains allelic diversity at MHC in vertebrates^34^,^35^ and acts in either an overdominant or frequency-dependent fashion^36^,^37^. Protein-coding sequences are often subject to purifying selection due to constraints on function, with positive selection operating on a small number of non-synonymous sites^38^. As the MHC *class II* molecule identifies and binds to continually changing array of foreign peptides, positive selection is expected in parts of the PBR^31^. In fact, some of the musk deer residues under positive selection (i.e. amino acid position 11), were the same as those observed by Schaschl *et al.*^31^ that screened over a dozen ungulate species. Thus, collectively our analyses support a role for recombination and selection in the generation and maintenance of forest musk deer MHC diversity.

Given the conservation status of musk deer, what does the observed MHC diversity mean for their persistence and survival? Forest musk deer have seen a drastic decline in numbers over the past half-century and are classified as endangered. Poaching and habitat loss have been the main drivers^15^, and this has brought on additional issues associated with small or declining populations. In particular, a high rate of mortality caused by musk deer purulent disease^20^ has become a major problem in captive populations. Emaciation and malnutrition caused by parasites is another problem captive populations are facing. Li *et al.*^20^ found differing susceptibility associated with the disease according to MHC class II haplotype (in a sample size of 99 individuals, 53 with disease symptoms), with nearly all the *DRB* exon 2 diversity observed in that study also being observed in our captive population. This is useful information for selective breeding programs as it suggests the species potentially harbors the required diversity to fend off pathogens. While a more comprehensive screening of resistant and susceptible haplotypes is warranted given the small sample size of Li *et al.*^20^ and the novel alleles detected in this study, we argue that managers—under the working assumption that certain haplotypes might harbor disease resistance in this species—should consider maximizing MHC variation in their breeding pool. We also advocate for further screening of the Class I loci, especially given the viral nature of purulent disease^39^, as well as non-MHC immune genes^40^,^41^. An enhanced screening program should be obtainable given the number of animals in captivity and genome-enabled nature of musk deer: we therefore urge musk deer captive breeders and wildlife managers and veterinarians to work together in this regard.

## Methods

### Sampling and DNA preparation

Fifty-two forest musk deer were randomly selected from a musk deer breeding centre in Li county, Sichuan province, China (N 31.662°, E 102.810°). The breeding centre houses approximately 500 individuals. Fresh blood samples were collected in vacuum tubes with EDTA and then brought to the laboratory and stored at −80 °C. All samples were collected in strict compliance with the Chinese Wildlife Conservation Act. This study was approved by the Wildlife Protection Station of Li county. All surgery was performed with the help of a local veterinarian, and all efforts were made to minimize suffering. This study was also approved by the Sichuan Pianzaihuang Musk Deer Corporation, who managed the musk deer we sampled. No other endangered or protected species were involved. Genomic DNA of forest musk deer was extracted from samples using the blood DNA extraction kit (Biomed technology Ltd.) according to the manufacturer’s protocol.

### PCR and MHC genotyping

The entire Exon 2 of the *DRB* gene was amplified with polymerase chain reaction (PCR) using primers LA31: 5′-GATGGATCCTCTCTCTGCAGCACATTTCCT; and LA32: 5′-CTTGAATTCGCGTCACCTCGCCGCTG^42^. This pair of primers was chosen from those previously designed bovine oligos. We assume a high degree of synteny based on the close phylogenetic relationship between Bovidae and Muschidae revealed by Hassanin and Douzery^43^. The amplification reaction was performed in 30 μl and contained 50 to 100 ng of genomic DNA, 15 μl of 2 × Taq PCR Master Mix and 0.5 μM forward and reverse primers. The PCR cycling parameters were as follows: denaturation at 94 °C for 5 minutes, 34 cycles of 94 °C for 30 seconds, 50 °C for 30 seconds, 72 °C for 45 seconds, and a final extension at 72 °C for 8 minutes. PCR products were visually assessed on a 1% agarose gel, and purified using the Axygen purification kit. The purified products were directly sequenced on an ABI PRISM 3730 XL DNA Sequencer.

Visual examination of the chromatograms showed that for the some individuals, multiple exon sequences were present suggesting multiple alleles or loci were present. For non-homogenous amplicons (i.e. samples with multiple sequences), PCR products were cloned. Here, heterozygous amplicons were cloned into the PMD18-T vector (Takara Bio Inc.) and transformed into DH5α competent cells following manufacturer’s protocol. A minimum of 16 transformed sub-clones were picked for each individual and then subjected to a PCR test to verify the insert size. Here, each 10 μl reaction mixture contained 2 × Taq PCR Master Mix, 0.5 μM specific forward and reverse primers under the following thermocycling conditions: 95 °C for 5 minutes, 33 cycles of denaturation at 94 °C for 30 seconds, annealing at 57 °C for 30 seconds, elongation at 72 °C for 45 seconds and a final extension at 72 °C for 10 minutes. Inserts with an appropriate length (~300 bp), assessed by eye on a 1% agarose gel, were then sequenced using the plasmid primers (M13).

Cloned sequences were compared within individuals and then checked for consistency by comparison with the sequences derived from direct sequencing of the amplicons. Additional subclones were sequenced when not all heterozygous sites (multi-peaks at the same site) observed in the direct sequencing were obtained (sequencing between 4 to 21 subclones were required). To ensure allele calls were not spurious or the result of sequencing error, each haplotype obtained for each individual had to be observed in two separate clonal sequences. Possible chimeras produced in the process of PCR were identified by comparison within individuals and then removed from the sequence pool. Nomenclature of forest musk deer *DRB* alleles followed Klein *et al.*^44^, henceforth referred to as *Mobe DRB*.

### Sequence analysis of polymorphism

Sequence alignment and translation were performed using MEGA 5^45^. The putative peptide-binding residues (PBR) were identified based on human HLA molecules^21^. The best-fitting nucleotide substitution model and the corresponding transition/transversion ratio (R) were determined by MEGA with the selected model based on the lowest BIC scores (Bayesian Information Criterion). A maximum likelihood (ML) phylogenetic tree was constructed using MEGA 5.0 and confidence assessed with one thousand bootstrap replicates. We also constructed a phylogenetic tree using MrBayes 3.2^46^ and the following criteria: 1,000,000 generations, with tree sampling every 10 generations and burn-in after 25,000 trees. DnaSP 5^47^ was used to calculate sequence polymorphism indices, including average number of nucleotide differences (k), number of segregating sites (S), and the nucleotide diversity per site (*π*) calculated for the whole sequence (*π*_total_), for non-synonymous sites (*π*_*n*_) and for synonymous sites (*π*_s_).

### Test for intragenic recombination

We employed an array of methods to detect recombination in order to minimize false positives^48^. First, we used the RDP3 package^49^, which employs a variety of methods (Table S1) to detect recombination breakpoint locations: GENECONV examines only silent polymorphic sites and is not strongly influenced by mutation variation or selective effects; MAXCHI identifies crossover points that maximize the difference between the proportions of polymorphic sites occupied by the same and by different bases, before and after the crossover; CHIMAERA uses variable sites to evaluate any possible combination of three sequences; RDP examines all possible triplets of sequences in a sliding window approach to estimate the probability of a recombination event; 3SEQ identifies variable sites that support different partitions of the data^47^. We used an alpha value of 0.05 in these analyses to determine significance. Population scaled recombination rate ρ and mutation rate θ were obtained by using LDhat recombination rate scan^50^. Finally, the GARD method^51^ was employed to search for possible recombination partitions.

### Detecting selection

We estimated the rate of non-synonymous (dN) and synonymous (dS) substitutions among all pairwise comparisons of *Mobe DRB* exon and corresponding bovine *DRB3* alleles using DnaSP. A sliding-window analysis of *π* and Tajima’s D was performed using a window size of 5 bp and a step size of 2 bp for *Mobe DRB* alleles. Coalescent simulations with 1000 replicates were run to test window analysis significance of Tajima’s D value. Metrics were also estimated for the entire exon, PBR and non-PBR designations, respectively. Statistical significance was tested using T-tests implemented in SPSS 19.

We used site-specific models of codon evolution available in the package PAML^52^ to assess the selection. Likelihood values were compared between each pair of models: M0 (one ratio) and M3 (discrete), M1 (nearly neutral) versus M2 (positive selection), and M7 (nearly neutral with the beta distribution) versus M8 (positive selection with the beta distribution). Likelihood ratio tests (LRTs) were carried out for the six models. A Bayesian approach implemented in CODEML (part of PAML) was used to identify residues under positive selection in the MHC *class II DRB* sequences. It should be noted that most traditional methods of detecting selection assume a single lineage and can be misleading in the presence of recombination^53^. The GARD approach avoids this by screening all alignments to locate all non-recombinant fragments (partitions), and allows each partition to have its own phylogenetic history. Thus the GARD results were incorporated into selection analyses so as to account for the presence of recombination. Three methods, single-likelihood ancestor counting (SLAC), fixed effects likelihood (FEL) and random effects likelihood (REL) analyses, were run on *Mobe DRB* alleles to detect sites under selection. Briefly, the SLAC compares the observed ratio of non-synonymous and synonymous substitutions using a maximum likelihood reconstruction of ancestral codon states; FEL infers model parameters shared by all sites (e.g., branch lengths) using the entire alignment and then fits dS and dN rates individually at every site; REL allows both synonymous and non-synonymous substitution rates to vary among sites^54^.

## Additional Information

**Accession codes:** Sequence data from this article can be found in the GenBank under the accession numbers: KP763631-KP763636, KP835297.

**How to cite this article**: Cai, R. *et al.* Recombination and selection in the major histocompatibility complex of the endangered forest musk deer (*Moschus berezovskii*). *Sci. Rep.* **5**, 17285; doi: 10.1038/srep17285 (2015).

## Supplementary Material

Supplementary Information

## Figures and Tables

**Table 1:** Sequence diversity of the 7 *Mobe DRB* haplotypes observed in this study.

Locus	k	S	*π*_total_	*π*_n_	*π*_s_	Tajima’s D (sig. test)
*BoLA DRB3*	17.41	55	0.07	0.08	0.05	2.41 (p < 0.05)
*Mobe DRB*	13.54	43	0.06	0.05	0.06	2.01 (0.10 > p > 0.05)

Samples from yellow cattle (*BoLA*) are included for comparison. Statistics are: k—average number of nucleotide difference; S—the number of segregating sites; π_total_—the mean number of nucleotide differences per site; π_n_—average pairwise differences calculated on non-synonymous sites; π_s_—average pairwise differences based on synonymous sites.

**Table 2:** Relative rates of non-synonymous (dN) and synonymous (dS) substitutions (with standard errors) calculated for bovine and *Mobe DRB* exon 2, averaged over all sites, the peptide binding region (PBR) and non-PBR.

Gene	*Mobe DRB* exon2			BoLA *DRB3* exon2		
	Overall	PBR	nonPBR	Overall	PBR	nonPBR
dS (SE)	0.10 (0.02)	0.14 (0.03)	0.08 (0.01)	0.06 (0.002)	0.14 (0.002)	0.03 (0.002)
dN (SE)	0.08 (0.01)	0.27 (0.02)	0.03 (0.003)	0.09 (0.003)	0.26 (0.01)	0.04 (0.001)
dN/dS	0.80	1.93	0.38	1.50	1.86	1.33
Sig. test	t = 1.052, p > 0.05	t = −7.65, P < 0.01	t = 5.59, p < 0.01	t = −13.83, p < 0.01	t = −12.96, P < 0.01	t = −4.09, P < 0.01

Statistical significance was tested using pair-wise T-tests.

**Table 3:** Number of MHC DRB alleles obtained from different species.

Species	Forest musk deer (this paper)	Spanish ibex^23^	Chamois^24^	Arabian oryx^25^	North American mountain goat^26^	White-tailed Deer^27^	Bighorn sheep^28^
Number of MHC *DRB* allels	7	6	7	3	2	15	21
Population sampled	52	43	18	57	212	150	213

## References

[b1] Sommer S. Effects of habitat fragmentation and changes of dispersal behaviour after a recent population decline on the genetic variability of noncoding and coding DNA of a monogamous Malagasy rodent. Mol Ecol. 12, 2845–2851, 10.1046/j.1365-294X.2003.01906.x (2003). 12969486

[b2] Kohn M. H., Murphy W. J., Ostrander E. A. & Wayne R. K. Genomics and conservation genetics. Trends Ecol Evol. 21, 629–637, 10.1016/j.tree.2006.08.001 (2006). 16908089

[b3] Bonin A., Nicole F., Pompanon F., Miaud C. & Taberlet P. Population adaptive index: A new method to help measure intraspecific genetic diversity and prioritize populations for conservation. Conserv Biol. 21, 697–708, 10.1111/j.1523-1739.2007.00685.x (2007). 17531048

[b4] Sommer S. The importance of immune gene variability (MHC) in evolutionary ecology and conservation. Front Zool. 2, 16, 10.1186/1742-9994-2-16 (2005). 16242022 PMC1282567

[b5] Piertney S. B. & Oliver M. K. The evolutionary ecology of the major histocompatibility complex. Heredity 96, 7–21, 10.1038/sj.hdy.6800724 (2006). 16094301

[b6] Schwartz M. K., Luikart G. & Waples R. S. Genetic monitoring as a promising tool for conservation and management. Trends Ecol Evol. 22, 25–33, 10.1016/j.tree.2006.08.009 (2007). 16962204

[b7] Sutton J. T., Nakagawa S., Robertson B. C. & Jamieson I. G. Disentangling the roles of natural selection and genetic drift in shaping variation at MHC immunity genes. Mol Ecol. 20, 4408–4420, 10.1111/j.1365-294X.2011.05292.x (2011). 21981032

[b8] Dukkipati V. S. R., Blair H. T., Garrick D. J. & Murray A. ‘Ovar-Mhc’—ovine major histocompatibility complex: structure and gene polymorphisms. Genet Mol Res. 5, 585–608 (2006). 17183471

[b9] Takada T. *et al.* Analysis of goat MHC class II DRA and DRB genes: Identification of the expressed gene and new DRB alleles. Immunogenetics 48, 408–412, 10.1007/s002510050452 (1998). 9799337

[b10] Nei M., Gu X. & Sitnikova T. Evolution by the birth-and-death process in multigene families of the vertebrate immune system. Proc Natl Acad Sci USA 94, 7799–7806, 10.1073/pnas.94.15.7799 (1997). 9223266 PMC33709

[b11] Andersson L. & Mikko S. Generation of MHC class II diversity by intra- and intergenic recombination. Immunol Rev. 143, 5–12, 10.1111/j.1600-065X.1995.tb00667.x (1995). 7558082

[b12] Gyllensten U. B., Sundvall M. & Erlich H. A. Allelic diversity is generated by intraexon sequence exchange at the *DRB*1 locus of primates. Proc Natl Acad Sci USA 88, 3686–3690 (1991). 2023919 10.1073/pnas.88.9.3686 PMC51517

[b13] Yi S., Summers T. J., Pearson N. M. & Li W. H. Recombination has little effect on the rate of sequence divergence in pseudoautosomal boundary 1among humans and great apes. Genome Res. 14, 37–43 (2004). 14672979 10.1101/gr.1777204 PMC314274

[b14] Gilpin M. & Wills C. MHC and captive breeding: a rebuttal. Conserv Biol. 5, 554–555, 10.1111/j.1523-1739.1991.tb00368.x (1991).

[b15] Hughes A. L. MHC polymorphisms and the design of captive breeding programs. Conserv Biol. 5, 249–251 (1991).

[b16] Wang Y. & Harris R. B. *Moschus berezovskii*. *The IUCN Red List of Threatened Species.* (2008) Available at: http://dx.doi.org/10.2305/IUCN.UK.2008.RLTS.T13894A4362437.en. (Accessed: 29th September 2015).

[b17] Yang Q., Meng X., Xia L. & Feng Z. Conservation status and causes of decline of musk deer (*Moschus* spp.) in China. Biol Conserv. 109, 333–342, 10.1016/S0006-3207(02)00159-3 (2003).

[b18] Li L. H. *et al.* The Status of captive population of musk deer and analysis of its farming development in China. Sichuan Journal of Zoology 31, 492–496, Chinese (2012).

[b19] Li L., Zhu Y., Ge Y. F. & Wan Q. H. Characterization of major histocompatibility complex *DRA* and *DRB* genes of the forest musk deer (*Moschus berezovskii*). Chinese Sci Bull. 58, 2191–2197, 10.1007/s11434-012-5581-5 (2013).

[b20] Li L., Wang B. B., Ge Y. F. & Wan Q. H. Major histocompatibility complex class II polymorphisms in forest musk deer (*Moschus berezovskii*) and their probable association with purulent disease. Int J Immunogenet. 41, 401–412, 10.1111/iji.12135 (2014). 25053118

[b21] Brown J. H. *et al.* Three-dimensional structure of the human class II histocompatibility antigen HLA-*DR*1. Nature 364, 33–39 (1993). 8316295 10.1038/364033a0

[b22] Liu H. Y. The diagnosis and treatment of the purulent disease of musk deer. J Southwest Uni Sci Tech. 19, 99–101, Chinese (2004).

[b23] Mainguy J., Worley K., Côté S. D. & Coltman D. W. Low MHC *DRB* class II diversity in the mountain goat: Past bottlenecks and possible role of pathogens and parasites. Conserv Genet. 8, 885–891, 10.1007/s10592-006-9243-5 (2007).

[b24] Amills M. *et al.* Low diversity in the major histocompatibility complex class II *DRB*1 gene of the Spanish ibex, Capra pyrenaica. Heredity 93, 266–272, 10.1038/sj.hdy.6800499 (2004). 15241456

[b25] Schaschl H., Suchentrunk F., Hammer S. & Goodman S. J. Recombination and the origin of sequence diversity in the *DRB* MHC class II locus in chamois (*Rupicapra* spp.). Immunogenetics 57, 108–115, 10.1007/s00251-005-0784-4 (2005). 15756546

[b26] Hedrick P. W., Parker K. M., Gutiérrez-Espeleta G. A., Rattink A. & Lievers K. Major histocompatibility complex variation in the Arabian oryx. Evolution 54, 2145–2151, 10.1111/j.0014-3820.2000.tb01256.x (2000). 11209789

[b27] Shafer A. B. A., Fan C. W., Côté S. D. & Coltman D. W. (Lack of) genetic diversity in immune genes predates glacial isolation in the north american mountain goat (*Oreamnos americanus*). J Hered. 103, 371–379, 10.1093/jhered/esr138 (2012). 22268162

[b28] Van Den Bussche R. A., Hoofer S. R. & Lochmiller R. L. Characterization of Mhc-*DRB* allelic diversity in white-tailed deer (*Odocoileus virginianus*) provides insight into Mhc-*DRB* allelic evolution within Cervidae. Immunogenetics 49, 429–437, 10.1007/s002510050516 (1999). 10199919

[b29] Gutierrez-Espeleta G. A., Hedrick P. W., Kalinowski S. T., Garrigan D. & Boyce W. M. Is the decline of desert bighorn sheep from infectious disease the result of low MHC variation? Heredity 86, 439–450, 10.1046/j.1365-2540.2001.00853.x (2001). 11520344

[b30] Mikko S. & Andersson L. Extensive MHC class II DRB3 diversity in African and European cattle. Immunogenetics 42, 408–413, 10.1007/BF00179403 (1995). 7590975

[b31] Schaschl H., Wandeler P., Suchentrunk F., Obexer-Ruff G. & Goodman S. J. Selection and recombination drive the evolution of MHC class II *DRB* diversity in ungulates. Heredity 97, 427–437, 10.1038/sj.hdy.6800892 (2006). 16941019

[b32] Mikko S., Lewin H. A. & Andersson L. A. phylogenetic analysis of cattle *DRB*3 alleles with a deletion of codon 65. Immunogenetics 47, 23–29, 10.1007/s002510050322 (1997). 9382917

[b33] Radwan J., Biedrzycka A. & Babik W. Does reduced MHC diversity decrease viability of vertebrate populations? Biol Conserv. 143, 537–544, 10.1016/j.biocon.2009.07.026 (2010). PMC7092871 32226082

[b34] Aguilar A. *et al.* High MHC diversity maintained by balancing selection in an otherwise genetically monomorphic mammal. Proc Natl Acad Sci USA 101, 3490–3494, 10.1073/pnas.0306582101 (2004). 14990802 PMC373489

[b35] Van Oosterhout C. *et al.* Balancing selection, random genetic drift, and genetic variation at the major histocompatibility complex in two wild populations of guppies (*Poecilia reticulata*). Evolution 60, 2562–2574, 10.1554/06-286.1 (2006). 17263117

[b36] Hedrick P. W. & Thomson G. Evidence for balancing selection at HLA. Genetics 104, 449–456 (1983). 6884768 10.1093/genetics/104.3.449 PMC1202087

[b37] Hedrick P. W. Pathogen resistance and genetic variation at MHC loci. Evolution 56, 1902–1908, 10.1111/j.0014-3820.2002.tb00116.x (2002). 12449477

[b38] Resch A. M. *et al.* Widespread positive selection in synonymous sites of mammalian genes. Mol Biol Evol. 24, 1821–1831, 10.1093/molbev/msm100 (2007). 17522087 PMC2632937

[b39] Luo Y. *et al.* Physicochemical properties of musk deer pneumonia and purulent disease viruses. Animal Husbandry and Feed Science 2, 37–40, Chinese (2010).

[b40] Acevedo-Whitehouse K. & Cunningham A. A. Is MHC enough for understanding wildlife immunogenetics? Trends Ecol Evol. 21, 433–438 (2006). 16764966 10.1016/j.tree.2006.05.010

[b41] Quéméré E. *et al.* Immunogenetic heterogeneity in a widespread ungulate: the European roe deer (*Capreolu*s *capreolus*). Mol Ecol. 15, 3873–3887, 10.1111/mec.13292 (2015). 26120040

[b42] Sigurdardóttir S., Borsch C., Gustafsson K. & Andersson L. Cloning and sequence analysis of 14 *DRB* alleles of the bovine major histocompatibility complex by using the polymerase chain reaction. Anim Genet. 22, 199–209 (1991). 1928826 10.1111/j.1365-2052.1991.tb00670.x

[b43] Hassanin A. & Douzery E. J. P. Molecular and morphological phylogenies of ruminantia and the alternative position of the moschidae. Syst Biol. 52, 206–228, 10.1080/10635150390192726 (2003). 12746147

[b44] Klein J. *et al.* Nomenclature for the major histocompatibility complexes of different species: a proposal. Immunogenetics 31, 217–219, 10.1007/BF00204890 (1990). 2329006

[b45] Tamura K. *et al.* MEGA5: Molecular evolutionary genetics analysis using maximum likelihood, evolutionary distance, and maximum parsimony methods. Mol Biol Evol. 28, 2731–2739, 10.1093/molbev/msr121 (2011). 21546353 PMC3203626

[b46] Ronquist F. *et al.* Mrbayes 3.2: Efficient bayesian phylogenetic inference and model choice across a large model space. Syst Biol. 61, 539–542, 10.1093/sysbio/sys029 (2012). 22357727 PMC3329765

[b47] Librado P. & Rozas J. DnaSP v5: A software for comprehensive analysis of DNA polymorphism data. Bioinformatics 25, 1451–1452, 10.1093/bioinformatics/btp187 (2009). 19346325

[b48] Posada D. & Crandall K. A. Evaluation of methods for detecting recombination from DNA sequences: computer simulations. Proc Natl Acad Sci USA 98, 13757–13762 (2001). 11717435 10.1073/pnas.241370698 PMC61114

[b49] Martin D. P. *et al.* RDP3: A flexible and fast computer program for analyzing recombination. Bioinformatics 26, 2462–2463, 10.1093/bioinformatics/btq467 (2010). 20798170 PMC2944210

[b50] McVean G., Awadalla P. & Fearnhead P. A coalescent-based method for detecting and estimatig recombination from gene sequences. Genetics 160, 1231–1241 (2002). 11901136 10.1093/genetics/160.3.1231 PMC1462015

[b51] McVean G. A. T. *et al.* The fine-scale structure of recombination rate variation in the human genome. Science (New York, NY) 304, 581–584, 10.1126/science.1092500 (2004). 15105499

[b52] Yang Z. PAML 4: Phylogenetic analysis by maximum likelihood. Mol Biol Evol. 24, 1586–1591, 10.1093/molbev/msm088 (2007). 17483113

[b53] Pond S. L. K., Posada D., Gravenor M. B., Woelk C. H. & Frost S. D. W. Automated phylogenetic detection of recombination using a genetic algorithm. Mol Biol Evol. 23, 1891–1901, 10.1093/molbev/msl051 (2006). 16818476

[b54] Sorhannus U. & Pond S. L. K. Evidence for positive selection on a sexual reproduction gene in the diatom genus *Thalassiosira* (Bacillariophyta). J Mol Evol. 63, 231–239, 10.1007/s00239-006-0016-z (2006). 16830090

