# Supplementary material for: Recombination and selection in the major histocompatibility complex of the endangered forest musk deer (Moschus berezovskii)
Source: Sci Rep. 2015 Nov 25;5:17285. doi: 10.1038/srep17285 (PMC4658564; doi:10.1038/srep17285)
Supplement: Supplementary Information [file srep17285-s1.doc]

**Recombination and selection in the major histocompatibility complex of the endangered forest musk deer (*Moschus berezovskii*)**

Ruibo Cai^1^, Aaron B.A. Shafer^2^, Alice Laguardia^1^, Zhenzhen Lin^3^,

Shuqiang Liu^1^, Defu Hu^1*^

1. College of Nature Conservation, Beijing Forestry University, China
2. Department of Ecology and Genetics, Uppsala University, Sweden
3. Key Laboratory of Animal Ecology and Conservation Biology, Institute of Zoology, Chinese Academy of Sciences, Beijing, China

^*^[hudf@bjfu.edu.cn](mailto:hudf@bjfu.edu.cn)

**Table S1. Recombination events detected by various methods in the RDP3 program.** * means the actual breakpoint position is undetermined. NS is short for “not significant”.

| Recombination Event Number | Breakpoint Positions in alignment | | Recombinant Sequence(s) | Minor Parental Sequence(s) | Major Parental Sequence(s) | Detection methods | | | | |
| --- | --- | --- | --- | --- | --- | --- | --- | --- | --- | --- |
|  | Begin | End |  |  |  | RDP | GENECONV | Maxchi | Chimaera | 3Seq |
| 1 | 1* | 9 | Mobe-DRB04 | Mobe-DRB01 | Mobe-DRB05 | NS | NS | NS | NS | 0.001 |
|  |  |  |  |  |  |  |  |  |  |  |
| 2 | 42* | 171* | Mobe-DRB04 | Mobe-DRB07 | Unknown (Mobe-DRB01) | 0.006 | NS | 0.001 | NS | 0.002 |
|  |  |  |  | Mobe-DRB03 | Unknown(Mobe-DRB02) |  |  |  |  |  |
|  |  |  |  | Mobe-DRB05 | Unknown(Mobe-DRB06) |  |  |  |  |  |
|  |  |  |  |  |  |  |  |  |  |  |
| 3 | 87* | 221 | Mobe-DRB02 | Mobe-DRB05 | Mobe-DRB01 | 0.013 | 0.042 | 0.001 | 0.005 | 0.004 |
|  |  |  | Mobe-DRB06 |  |  |  |  |  |  |  |
|  |  |  |  |  |  |  |  |  |  |  |
| 4 | 29* | 86* | Mobe-DRB05 | Unknown (Mobe-DRB01) | Mobe-DRB06 | NS | NS | NS | NS | 0.024 |
|  |  |  |  |  | Mobe-DRB02 |  |  |  |  |  |
|  |  |  |  |  |  |  |  |  |  |  |

**Table S2.** Recombination events detected by various methods implemented in RDP3 package.

| Methods | Unique events | Recombination signals |
| --- | --- | --- |
| RDP | 2 | 3 |
| GENECONV | 1 | 1 |
| MaxChi | 2 | 3 |
| Chimaera | 1 | 1 |
| 3seq | 4 | 3 |

**Table S3. Breakpoints detected by GARD method**

| Breakpoint | LHS p-value | RHS P-value | significance |
| --- | --- | --- | --- |
| 33 | 0.002 | 0.018 | <0.01 |
| 174 | 0.219 | 0.020 | N.S |

**Table S4. Tests for positive selection using different site models. The residues with a line below are located in PBR.** LnL is the log-likelihood value. Sites inferred to be under positive selection are given at the 95% (*) and 99% (**) confidence interval level.

| Model | abs(△lnL) | p-value | Positively selected sites |
| --- | --- | --- | --- |
| M0 vs M3 | 44.941 | p<0.001 | - |
| M1vs M2 | 17.628 | p<0.001 | 9^**^,10,11^**^,25,28^*^,37,61^*^,71^**^,74^**^,78,86 |
| M7 vs M8 | 17.642 | p<0.001 | 9^**^,10^*^,11^**^,25,28^**^,37^*^,61^**^,71^**^,74^**^,78,86^*^ |

**Table S5. Positively (PSS) and negatively (NSS) selected sites detected by REL, FEL and SLAC methods (* indicates the significance level with a posterior probability of ≥ 0.95, and ** indicates ≥ 0.99. The residues with a line below are located in PBR).**

|  | **PSS** | | | | | |  | | **NSS** | | |
| --- | --- | --- | --- | --- | --- | --- | --- | --- | --- | --- | --- |
|  | **REL** | **FEL** | | | **SLAC** | |  | | **REL** | **FEL** | **SLAC** |
| *Mobe*  *DRB*  exon2 | 9**,10**,11**,  28**,61**,  71**,74**,86** | | 11*,86 | 11 | |  | | 13**,19**,33**  ,48**,51**,  63**,77**,84**,  85** | | 13*,19*,33**,  51,63*,77 | 13,19,26,  33,63 |
